# Bridging and bonding interactions in higher education: social capital and students’ academic and professional identity formation

**DOI:** 10.3389/fpsyg.2015.00126

**Published:** 2015-02-13

**Authors:** Dorthe H. Jensen, Jolanda Jetten

**Affiliations:** ^1^Social and Personality Psychology, Department of Psychology and Behavioral Sciences, School of Business and Social Sciences, Aarhus University, Aarhus, Denmark; ^2^School of Psychology, The University of Queensland, Brisbane, QLD, Australia

**Keywords:** identity formation, academic identity, professional identity, self-concept, bridging social capital, bonding social capital

## Abstract

It is increasingly recognized that graduates’ achievements depend in important ways on their opportunities to develop an academic and a professional identity during their studies. Previous research has shown that students’ socio-economic status (SES) and social capital prior to entering university affects their ability to obtain these identities in higher education. However, what is less well understood is whether social capital that is built during university studies shapes identity development, and if so, whether the social capital gained during university years impacts on academic and professional identity differently. In a qualitative study, we interviewed 26 Danish and 11 Australian university students about their social interaction experiences, their opportunities to develop bonding capital as well as bridging capital, and their academic and professional identity. Findings show that while bonding social capital with co-students facilitated academic identity formation, such social capital does not lead to professional identity development. We also found that the development of bridging social capital with educators facilitated students’ professional identity formation. However, bonding social capital among students stood in the way of participating in bridging interaction with educators, thereby further hindering professional identity formation. Finally, while students’ parental background did not affect the perceived difficulty of forming professional identity, there was a tendency for students from lower SES backgrounds to be more likely to make internal attributions while those from higher SES backgrounds were more likely to make external attributions for the failure to develop professional identity. Results point to the importance of creating opportunities for social interaction with educators at university because this facilitates the generation of bridging social capital, which, in turn, is essential for students’ professional identity development.

## INTRODUCTION

In today’s society, graduates’ economic success is shaped in important ways by educational competencies and individuals’ ability to flourish in complex work environments (Goh and Lee, 2008). As a result, researchers and university policy makers alike are increasingly interested in the integration between academic skill development and workplace needs and, more generally, the development of workplace skills while students are at university. This has led to a focus on students’ opportunities to develop not only academic but also professional identities during their studies (Farrell, 1990; Pascarella and Terenzini, 2005; Scanlon et al., 2007; Trede et al., 2012; Komarraju et al., 2010). The importance of developing professional identities as part of the curriculum puts pressure on universities to provide opportunities for students, such as work-integrated learning, that ensures optimal preparation for future workforce conditions (Barnett, 1994; Reid et al., 2008).

However, it is also clear that not all students benefit equally from opportunities to develop identity at university. Previous correlational research has shown that the students who benefit most from these opportunities are those from higher socio-economic status (SES) who, prior to entering university, have more social capital than their lower SES counterparts—social capital that forms an important building block for the development of these identities in higher education (Rendón, 1994, 2002; Hurtado and Carter, 1997; Nora et al., 2005; Freeman et al., 2007; Jetten et al., 2007; Pittman and Richmond, 2007; Iyer et al., 2008). However, social capital is not fixed or set in stone and new social capital is formed while students are at university. With the focus of the research on social capital formed before university entry, it is unclear whether social capital that is built after arrival at university affects identity development. Furthermore, if such ongoing social capital development is important for identity formation, it is unclear whether different forms of social capital gained in higher education affect the development of academic and professional identity in different ways.

This research sets out to explore whether students experience their social interactions at university as supportive in gaining different forms of social capital. A second aim is to gain insight into whether social capital accumulated at university facilitates or hinders the development and consolidation of academic and professional identity. A further aim was to examine if the ability to develop identity and social capital at university is influenced by students’ SES (measured as parents’ educational background).

### SOCIAL CAPITAL AND ACADEMIC ACHIEVEMENT

Social capital is defined as the value derived from membership in social groups, social networks or institutions. Such membership gives individuals access to resources and collective understanding. Putnam (2000) describes different forms of social capital, whereby social capital can be derived from shared experience, cultural norms, or shared purposes. Research has shown that a university student’s social capital derived from family background affects their academic achievement outcomes in relation to preparation and persistence in higher education (Bourdieu, 1986; Rendón et al., 2000; Tierney, 2000; Horvat et al., 2003; Anderson, 2005; Ream, 2005). The difficulties arising from incompatibility of students’ social capital background and higher educational achievement is well documented. For example, low-income, first-generation, or minority students are less likely to attend or finish university than their more privileged peers with similar academic qualifications (Simmons, 2011). One of the reasons for this is that for some students, attending university is incompatible with their family background and this has been found to predict poorer outcomes in the long term (Iyer et al., 2008) and that makes it more difficult for them to prepare for university (Horvat et al., 2003; Ream, 2005), and to access university (Tierney and Jun, 2001; Anderson, 2005; Kim and Schneider, 2005; Perna and Titus, 2005; Simmons, 2011). The lack of social capital also affects students’ choice of university major (Porter and Umbach, 2006), and their university experience more generally (Pascarella and Terenzini, 2005; Harper and Quaye, 2008).

Another reason that low-income, first generation or minority students are less likely to attend university arises from the fact that SES and social capital prior to entering university impacts students’ subsequent ability to obtain academic or professional identity in higher education (Hurtado and Carter, 1997; Jetten et al., 2007; Scanlon et al., 2007; Iyer et al., 2008). Higher social capital—in particular the type of capital that affects development of an academic identity—makes identity change easier, as those with greater social capital view the transition to university as a “normal” part of their lives. This, because entering university is compatible for these students with their existing identity network (Hurtado and Carter, 1997; Freeman et al., 2007; Jetten et al., 2007; Iyer et al., 2008). Furthermore, students from higher SES backgrounds have access to a greater diversity of group memberships that can support identity transitions (Jetten et al., 2013). Students without the required social capital therefore find this identity work particularly challenging (Raffo and Reeves, 2000; Read et al., 2003; Gardner and Barnes, 2007; Scanlon et al., 2007). They often lack the understanding of the values, norms, and language in higher education as well as the support of family and friends that can ease such a transition and facilitate academic identity formation and high academic achievement (Rendón, 1994, 2002; Hurtado and Carter, 1997; Nora et al., 2005; Freeman et al., 2007; Pittman and Richmond, 2007; Scanlon et al., 2007).

We propose that a more fine-grained analysis of different types of social capital helps to unpack these processes further. Consistent with Putnam (1993, 2000) analysis, we distinguish between *bonding* and *bridging* social capital. Bonding social capital refers to the resources that individuals derive from relations with others and membership in social groups that allow for the perception of shared identity (Jetten et al., 2014). In groups that provide high levels of bonding capital, interactions are characterized by strong social ties, high social support and loyalty toward other members. In contrast, interactions that go across group boundaries, and therefore allows for the development of identities outside the in-group can form the basis of bridging capital. While the ties in bridging networks are not as strong as those that characterize bonding relations, the former form of capital provides access to information outside of the immediate network and allows for the development of identity (Putnam, 1993).

Therefore, depending on the nature of an individual’s social capital, different types of identity development can be facilitated (Raffo and Reeves, 2000). As we outline in further detail below, we predict that bonding capital facilitates the development of academic identity whereas bridging capital is essential for the formation and development of professional identity. Before further developing these predictions, it is important to first define academic and professional identity and to explain their link to academic achievement.

### ACADEMIC AND PROFESSIONAL IDENTITY

In the higher educational literature, academic identity is defined as the extent to which students feel they belong to the greater academic community, students’ experience of personal academic worth and their visibility in the academic environment (Pascarella and Terenzini, 2005). Studies show that students’ academic self-concept, their academic identity and sense of belonging to the environment, are significantly related to their academic achievement (Chickering and Reisser, 1993; Lounsbury et al., 2005; Pascarella and Terenzini, 2005; Pasque and Murphy, 2005; Scanlon et al., 2007; Torres et al., 2009; Hughes, 2010). What is more, with the growing recognition of the importance of students being optimally prepared when they enter the workforce, there is an increasing pressure on students’ to develop not only academic skills but also know-how and operational skills related to their field (Barnett, 1994). Graduate achievement therefore depends significantly on students’ opportunities to develop both academic and professional identities during their studies (Murphy et al., 2009; Komarraju et al., 2010; Trede et al., 2012; Molinero and Pereira, 2013). Professional identity formation involves the development of an awareness of the values, responsibilities but also the personal resources that are essential in the future professional environment (Bruss and Kopala, 1993; Öhlén and Segesten, 1998; Vasile and Albu, 2011).

It is unclear whether different forms of social capital gained in higher education affect the development of academic and professional identity in different ways. We expect that students may derive bonding capital from interactions with peers at university. We predict that these interactions and the sense of belongingness that emerges from participation in group activities with peers particularly contributes to the development and formation of an academic identity because it helps students to understand the university environment and teaches them to successfully navigate this world. Consistent with this, there is a body of work that suggests that students inherit social capital from being in the academic environment and having social interactions with their fellow students (Bensimon, 2007; Scanlon et al., 2007).

Within universities, students will also interact with others with whom they do not necessarily share identity but these interactions form important building blocks for the formation of bridging capital. Consistent with this, some have pointed to the importance of interactions between students and educators that facilitate the process whereby students are able to view themselves as academics (Bensimon, 2007; Komarraju et al., 2010). Educators not only have an important role to play in academic identity formation, but they are also uniquely positioned to advise students on future job prospects and the required skills in the workforce and they can act as important role models—all of which contribute to the development of a professional identity.

However, given that students from lower SES backgrounds experience greater difficulty creating social capital than their higher SES counterparts, it remains to be examined whether these social inequalities carry over within the university context. In particular, we ask whether SES background affects the extent to which students are able to form bonding and bridging social capital at university but also the extent to which this facilitates the development of academic and professional identity. Based on previous research findings, we anticipated that students from lower SES backgrounds might be less likely to connect and form relationships with co-students. This leaves them isolated (Lawrence, 2001; Read et al., 2003) or excluded (Pargetter, 2000; Raffo and Reeves, 2000) and hinders the development of academic identity. Furthermore, given that lower SES students may feel less at home at university than their higher SES background counterparts because they perceive attending university as inconsistent with their family background (see Jetten et al., 2007; Iyer et al., 2008), we were open to the finding that they would be less likely to seek out educators’ help and advice and that this would negatively affect the formation of their professional identity.

### INSTITUTIONAL BARRIERS AND FACILITATORS

The way that universities are structured may affect the ease with which students perceive that there are opportunities to gain social capital. It could be argued that there are a number of reasons why such opportunities have declined over the last decades. First, universities are increasingly conceptualized as research institutions rather than teaching institutions (Perry and Allard, 2003) As a result of this, while the traditional model of scholarly training—for example the Oxbridge model—involved intense contact between a tutor and a small group of students, academic-student interaction, particularly face-to-face interaction, has reduced over the last decades (Scanlon et al., 2007; Torres, 2007). Second, campus life has become less lively because campuses are increasingly decentralized and a large proportion of students learn remotely (Bridges, 2000). Third, attending universities has become more expensive in many countries necessitating that students work to pay for their studies. As a result, students spend less time on campus and they have less time to engage in activities that facilitate social capital development and identity formation (Smith and Webster, 1997).

Despite this, considerable pedagogical effort is expended on providing rich learning environments (Kuh et al., 2006) that promote volunteering and participating in community activities, clubs, and social organizations (Astin, 1993; Tinto, 1993; Pascarella and Terenzini, 2005; Gardner and Barnes, 2007). However, it is unclear if these forms of social interaction are efficacious in developing the forms of social capital that provide for both academic and professional identity formation. It is also unclear whether students recognize opportunities that are provided by institutions to develop academic and professional identity as such. And, if they do recognize these opportunities, the question remains whether they perceive that there are barriers in making use of these opportunities. We suspect that the perception of barriers may explain why previous work has shown that students report lacking opportunities to develop academic identity and identity in their professional field (Farrell, 1990; Lapsley et al., 1990; McInnis and James, 1995; Freeman et al., 2007; Pittman and Richmond, 2007; Meeuwisse et al., 2010).

### THE PRESENT RESEARCH

In an attempt to uncover opportunities that can strengthen the processes of identity formation for students in higher education, we conducted a qualitative study examining the interplay between different forms of social capital (bridging and bonding capital) and different identity formations (academic and professional identity formation). Specifically, we examined whether students experience their social interactions at university as supporting them in gaining bridging and bonding forms of social capital. A second aim is to get insight into whether social capital gained at university facilitates or hinders the development and consolidation of academic and professional identity. A further aim was to examine if the students’ experiences were influenced by the parents’ educational background.

We conducted our research at two universities in two different countries: at the University of Aarhus (AU), Denmark and The University of Queensland (UQ), Australia. Both AU and UQ are in the top 100 ranking (http://www.au.dk/om/profil/ranking/) but the University of Aarhus is smaller (37,500 students with 3,400 international students) than The University of Queensland (47,000 students enrolled, with approximately 16,000 international students mostly from Asia). Students from both universities are mostly of traditional age, from middle-class families and they experience college in a university and student culture characterized by academic and personal support options, possibility of high involvement and a tradition of academic focus. Support options, include student counseling services, advising about study options and choices, and personal counseling and support focusing on students’ well-being at university. Additionally, a range of courses is available to help the student structure and improve their skills in reading and writing and manage IT programs or tools. The students attend lectures, seminars and instructing or tutoring classes. During lectures, an average of 200–400 students attend and the instructing classes or seminars average around 30 students per class. As lectures or seminars often include the lecturers’ own research, students often have options to get involved in research through internships or students assistant jobs, either paid or volunteering. The universities differ though in their program structure: in the first two undergraduate years, the courses at UQ are open and broad whereby students can select courses from different schools (e.g., psychology and law). At AU, students select their degree program upon entering university. To shed light on the way the broader structure affects responses, in all our analyses, we examined whether participants’ experiences differed by university. Despite the differences between the universities and the different educational contexts in these two countries, in the findings reported below, experiences across the two contexts were rather similar. We therefore do not explore the role of these contextual differences any further.

We examined our hypotheses using interpretative phenomenological analysis (IPA)—a constructivist epistemological approach (Lincoln and Guba, 1985). Because constructivism relies on a relativist ontology, which assumes multiple realities and a subjectivist epistemology (Denzin and Lincoln, 2000), this methodology allows us to gain an understanding in how participants create meaning and develop an understanding of self and identity (Guba and Lincoln, 1994; Schwandt, 1994) by sharing their experiences with the researcher (see Crotty, 1998; Chamaz, 2000). This approach is therefore optimally suited to assess our research questions that relate to the detailed examination of a particular phenomenon (students’ identity formation) as it is experienced and given meaning in the life-world (academic environment) of a particular person (student).

## MATERIALS AND METHODS

### PARTICIPANTS AND CONTEXT

We recruited participants with different family educational backgrounds, ages, course levels, and study programs. At AU in Denmark, 26 students (11 undergraduate and 15 Masters students, five men and 21 women, with an average age between 24 and 25, ranging from 19 to 43 years of age) were interviewed. At UQ in Australia, 11 students (nine undergraduate students, one Honors and one Masters student, six women and five men, with an average age of 21, ranging from 18 to 31 years of age) were interviewed.

Twenty-four participants were considered first generation students (including 11 students with two parents whose highest level of education was high school and 13 students with at least one parent with a vocational background as the highest level of education, of these 17 students were from AU and seven students were from UQ). Thirteen students had at least one parent with an academic background (nine students from AU and four students from UQ).

Participating students studied in both the natural and social sciences (from 8 different fields at AU: Psychology, Political Science, Pedagogical teaching development, Business and Economy, Nano Science, Odontology, Medicine and Molecular Medicine, and from 7 fields at UQ: Psychology, Business Administration, Speech Pathology, Social Work, Mathematics, Biology, and Chemistry).

### PROCEDURES

Data was collected at AU from October 2011 until June 2012, and at UQ in May and June 2013. All students volunteered to take part in one and a half hour semi-structured interviews and these interviews were carried out on campus in a relaxed atmosphere.

At the University of Queensland, ethical clearance was obtained from the school of psychology and the study was conducted within the guidelines of the National Statement on Ethical Conduct in Human Research. There are no ethical guidelines for conducting this type of qualitative interviewing in Denmark and, at this site, no clearance was obtained. In both samples, students were informed about the purpose of the study and gave consent prior to interviewing.

The interview focused on students’ lived educational experience. From these experiences, the meaning of and connections between four general concepts were investigated: bridging and bonding forms of social interactions (social capital), academic identity formation, professional identity formation, and academic achievement. In this way, we aimed to identify the dynamic between forms of social capital and academic and professional identity formation taking as a starting point the student’s own experience of their educational context.

The interview consisted of several parts. First, participants were encouraged to talk about their experiences before entering university. They were asked about their background, how much their family and friends expected university attendance, how they decided on their topic of study, and how they experienced the transition from school to university. The second part of the interview consisted of questions about experiences as a university student. Part one and two were intended to shed light on *earlier* and *present* experiences of being in an academic culture and their prior social capital. The third part of the interview asked about phenomenological experiences that had affected participants’ self-understanding, both as academics and as professionals and/or learners. Generally the questions were non-directive and open-ended. For example, participants were asked: “Tell me about an experience that has had an impact on how you see yourself now or in the future?”, “What happened in that situation?” and “Who was involved?”, or “Describe this form of interaction in your own words?”, “Why do you think that this experience had that effect?”, “How much did you think about it afterward?”, “How often do you have similar experiences?” This gave us information about experiences of social interaction that affected the student’s learning or academic and professional identity formation as well as detailed information on the nature of the social capital that was gained.

Sampling was carried out until a sufficiently diverse sample of interviews had been included and until no new topics emerged (determined both by theory and data, Guest et al., 2006). Interviews were audio-recorded and subsequently transcribed. Transcription involved documenting all interviews conducted in Denmark and parts of the interviews conducted in Australia including comments on expressed emotions during the interviews.

### ANALYTICAL TECHNIQUE

Data were analyzed using IPA. IPA is particularly useful when exploring dynamic topics such as sense-making, self and identity (Smith, 2004). This is because IPA is based on phenomenology and symbolic interactionism and holds “that human beings are not passive perceivers of an objective reality, but rather that they interpret and understand their world by formulating their own biographical stories into a form that makes sense to them” (Brocki and Wearden, 2006, pp. 87–88; see also Smith et al., 1995; Smith, 2004). The role of the interviewer in this process is to encourage elaborations that increase knowledge about the phenomena under investigation and the connections between recognition and identity formation. The aim of IPA is to explore the detailed processes of how participants make sense of their own experiences (Smith et al., 1997; Chapman and Smith, 2002). This was achieved by examining how participants accounted for the processes of interpretation that made their experiences understandable to them.

To benefit from the full sample, all interviews were manually analyzed (maximum variation sampling, Patton, 2002) by the first author following the procedural guidelines associated with IPA (Smith, 1996; Smith and Osborn, 2003; Brocki and Wearden, 2006). A first step involved a careful and close reading of the transcripts and recordings followed by a continuous cycle of reduction and interpretation focusing on broad themes followed by a more fine-grained comparison between interviews. In the second step in the analysis, all participant statements along with condensed comments relating to a specific theme were copied into separate theme documents. These theme documents were first subjected to an iterative process of rechecking interpretations followed by an axial coding process aimed at identifying connections between the themes. These connections were annotated to understand processes and “thickness” between themes, as in the number of occurrences of a specific connection (Smith, 2004). This allowed for the development of greater insight into how a process occurred and how a particular connection was made between a form of social interaction, academic identity, professional identity, and academic learning. In the second step, the analytical focus shifted from the experience of the individual to a focus on comparative experiences through the pooling of quotations and connections that were found between themes. In this process, the original data (or the student’s own words) were included in the documents to ensure that their meaning and experience was not lost when generalizing across individuals.

## FINDINGS

The results of this study will be presented in the following order. We start with an analysis of interactions with other students that affect bonding and bridging social capital and academic and professional identity formation. We then examine social interactions with educators that give rise to bridging social capital facilitating academic identity formation, followed by social interactions between students and educators in relation to bridging social capital that facilitated professional identity formation. Finally, we explore the perceived opportunities to develop bridging social capital. In all our analyses, we examine processes separately for students from lower and higher educational family backgrounds.

### SOCIAL INTERACTIONS WITH CO-STUDENTS AND BONDING AND BRIDGING SOCIAL CAPITAL

The most prominent and salient relation found among all participating students’ experiences was the relation between social interactions with co-students and gaining bonding social capital. However, looking more closely at this relationship, not all experiences of gaining bonding social capital with co-students were equally effective in facilitating academic and/or professional identity. The extracts below illustrate how students from both backgrounds all perceived that engagement in extra-curricular activities in particular were not useful for developing academic identity. We first present the extracts from students from lower educational family background.

At the beginning I was very interested in extra-curricular activities but as the semester went on it got more difficult to…like…being motivated to do that. I didn’t feel like I became closer to feeling like an academic by joining them, not many of these activities are related to the academics. It is just activities provided in the higher academic context, really, and that can be convenient, but I don’t find them to support my academic identity (Georga, Speech Pathology, Lower educational family background, UQ).

I don’t join the extra-curricular activities. This is not a place you go to find community or your identity. This is a place you go when you have that already (Miriam, Psychology, Lower educational family background, UQ).

As students from a lower educational family background indicated, they benefitted most from academic related interactions with other students.

I think that my academic identity benefits the most from activities related to the academic learning. Like when we discuss things in small groups and we talk about the topics and you can tell that you become better at discussing things over time. Then you feel more like an academic. Not only do I not relate being an academic to extra-curricular activities, I don’t really enjoy them either (Emilie, Medicine, Lower educational family background, AU).

I really need a study partner to discuss things with…here you can be open and explore things…also talk about the things that are difficult. If it is a good functional relationship you help each other in being confident about being an academic. I think this is so much more beneficial than joining other stuff. I really don’t feel like going to all that (Claudia, Medicine, Lower educational family background, AU).

These extra-curricular activities are hard to go to, as everybody seems to know each other or someone. You need to feel like you belong and feel safe in displaying that before you go. Discussing academics with a fellow students is much more supporting to my identity (Georga, Speech Pathology, Lower educational family background, UQ).

The following extracts describe experiences from students from a higher educational family background. Students from higher educational backgrounds seemed to enjoy extra-curricular activities more than students from a lower educational background but they too perceived these extra-curricular activities as not essential to their academic identity formation or their academic achievement. For example, students from higher educational backgrounds mentioned:

I like being part of something here. It provides safety in a way. That I’m part of something that I can be proud of, like a community feeling. But I don’t really attend all the extra-curricular activities that they provide. Even though our tutors suggest them. But what I need is more social relations that relate to my study. Because I need to feel that I’m on safe ground, seeing myself as an academic. I need interactions that can support me in that (Lea, Odontology, Higher educational family background, AU).

We do have lots of possibilities to go to parties or hangout in general, have fun. However, I feel that I need more activities that are field related to form my identity here. This is what we (students) really need to feel confident and feel like academics. But there aren’t that many of such activities in our field. Mostly you can just join extra-curricular activities about other things, like a party, sports or clubs of some sort (Mathew, Chemistry, Higher educational family background, UQ).

I prefer social interactions in the academic courses rather than in non-related activities. I just really benefit so much more from talking about the curriculum and us in that world, rather than going to parties or sports with co-students…that’s ok too…but to me, interaction related to the field is what helps me to feel like an academic (Nicole, Psychology, Higher educational family background, UQ).

I do enjoy extra-curricular activities. But that said I don’t really gain academic identity from it. What supports that is social bonding with a few students related to the course (Mathew, Chemistry, Higher educational family background, UQ).

We can be social from both extra-curricular activities and when we have discussions about the curriculum. As much as I enjoy doing sports or going to some event, this is not where I really support my academic identity. I have to be good in my field to feel that, and I only find out how well I do, when I discuss academic topics with others (Morten, Political Science and Government, Higher educational family background, AU).

In sum, the results showed that the social interactions related to academic learning rather than social interactions during extra-curricular activities were the interactions most closely related to a form of bonding social capital that facilitated academic identity formation. What we generally found was that students gained this beneficial bonding social capital from one-to-one social interactions with another student or within smaller group interactions when these interactions related to academic learning.

However, despite this, we found that students perceived that their interactions with other students were not providing high quality bonding capital that allowed them to successfully develop and build academic identity formation. Rather than developing their academic identity further, bonding capital only provided them with a sense of belonging. Even though this was important to make them feel at home at university and to persevere with their studies, it did not seem to make students feel confident about themselves as academics. In this study, students expressed insecurities about their academic position regardless of their level of seniority as students. What is more, students from lower educational backgrounds did not describe examples of interactions with co-students differently from their counterparts from higher educational backgrounds.

I just really think that we have support in each other as students – just having a few friends that all share this process. Then we can keep each other on our feet and talk each other out of dropping out. I think we have saved each other from doing that several times by just sharing our understandings and worrying about the academic learning (Nicole, Psychology, Higher educational family background, first year, UQ).

We share our academic reading and understandings. It is so rewarding to have something in common with other students. This is convincing you that you can do this. You don’t have to be the best… it just ensures you that you are not falling off the academic wagon (Emilie, Medicine, Lower educational family background, second year, AU).

I use social bonding with other students to share feelings, especially the difficult feelings, like admitting that this text was difficult for me, and then others agree they felt the same way. This way we support each other in believing that we will make it, like a sense of community that we will be ok staying at university (Theiss, Psychology, Lower educational family background, Third year, AU).

I interact with my study-partner, we try to keep each other at university, helping each other preparing for and passing exams. One-to-one has helped me, as we don’t have that many social things relating to the curriculum. I have tried to manage one semester without a study-partner, and that didn’t go well. It is important to support each other in staying at university. It helps when another person sees you as an academic (Claudia, Medicine. Lower educational family background, fourth year, AU).

The difficulties we have and the uncertainty that we feel, we handle by talking to each other about it. You can go for a long time thinking that you are the only one, until you finally have a talk with a co-student and find that she also feels that way. Then we help each other each time we feel lost with our studies (Lea, Odontology, Higher educational family background, fourth year, AU).

I believe that this group bonding that we have on some of the natural science courses like math and physics, it is really 100% what gets students through these courses. The others are there to pick you up on the days where you’re not sure if you’ll make it. They will keep you at university (Niels Nano Science, Lower educational family background, fifth year, AU).

In sum, students generally described social interactions with co-students as resembling bonding social capital that kept them on their feet in tackling their studies through mutual support and ensuring a feeling of belonging to the academic environment (Haslam et al., 2005). Interestingly too, while many students talked about the role of social interactions in helping them to develop bonding capital, they did not mention fellow students in relation to the development of bridging capital.

### BRIDGING SOCIAL CAPITAL WITH EDUCATORS AND IDENTITY FORMATION

Our results show that bridging interactions between students and educators facilitated academic identity formation. However, students only described a handful of such interactions and all students described their interactions with educators as more distant. The few examples we found representing bridging interaction between students and educators revealed no differences between students from lower and higher educational family background. For example, the following extracts represent descriptions of interactions by students from lower educational family background.

When I matter to my tutors, like when they interact with us to make sure that we understand, then it also makes me more confident that I will become one of them, you know…a skilled academic. Interacting with other students does not do that for me (Anthony, Social Work, Lower educational family background, UQ).

I have worked in a lab for an academic and we got to interact that way. Sensing that she invested time in me, wanting me to learn this, even if it was to really help her own project, it still made me feel like I was a good academic that had potential in this field (Gitte, Molecular Medicine, Lower educational family background, AU).

The following extracts are from students from a higher educational family background.

I have had a few interactions with an academic. She was really genuine and I felt that it made a difference to her if I understood her explanation about this topic. It made me feel like a talented student that she would go to this effort (Alexandria, Psychology, Higher educational family background, UQ).

I have only met one academic that actually talked to me. She gave me feedback on an exam and said that I had done some good work. That really helped me in viewing myself as an academic (Ira, Psychology, Higher educational family background, AU).

As can be seen in these extracts, students described certain characteristics that were attributed to them (i.e., talented student or skilled academic) during the few bridging interactions they had had and this facilitated academic identity formation.

### BRIDGING SOCIAL CAPITAL WITH EDUCATORS AND PROFESSIONAL IDENTITY FORMATION

We only found a few examples of interactions between students and educators representing bridging capital that facilitated the development of students’ professional identity. The extracts below illustrate how bridging interaction can build professional identity. The first extracts are from students from lower educational family backgrounds.

I find it really encouraging and inspiring when my lecturers are so helpful in explaining things, taking their time, either interacting during breaks or writing me an email. Well, when they have really good personalities, and try to help me understand, it is often putting the learning into a frame of what the field is about that is really helpful to hear them describe. I have ideas, but just as an outsider in a way, watching that world…they help me understand that world, so that I can become part of it. I am really grateful for that. Without that, although I might learn something, I don’t feel like I develop as a professional (Steven, Psychology, Lower educational family background, UQ).

Interaction with lecturers or professionals is really necessary for this learning to be integrated in you, so that you start seeing yourself as a person in this field, because then you understand the field and its values (Miriam, Psychology, Lower educational family background, UQ).

The following extracts are from students from a higher educational family background.

It is really important to me that the lecturers will interact with us, because that is the way we get this second dimension of learning …you know, understanding this in a context and also understanding ourselves in that context, how we are as professionals. Because they can explain to us about the field and how all this fits together. And I think that if someone is not interacting at all and things get complicated to understand because of this, I will just start using other sources… start watching other lectures from other universities if they are available online. If it’s just for the learning, I can shop around (Mathew, Chemistry, Higher educational family background, UQ).

I don’t understand so much about the future job in this, but if they (lecturers) interact with us, I get really inspired being with such bright people on a day-to-day basis. That really develops me as well. I start identifying with them (Morten, Political Science, Higher educational family background, AU).

### THE DOWNSIDE OF BONDING SOCIAL CAPITAL AMONG CO-STUDENTS

One of the reasons why students only rarely mentioned social interaction with educators is that bonding interactions with other students hindered seeking out bridging interactions that could facilitate academic and professional identity formation. That is, membership in groups that provided bonding capital was also associated with a strong student culture that prevented students from considering joining new groups and developing new relationships, such as with students at higher course levels or with educators. The extracts below are students’ descriptions of norms within the student group preventing students from asking questions or approaching educators. The extracts also show that students perceive educators as different from them and their student group. The following descriptions are from students from lower educational family backgrounds.

I look up to educators, but I don’t really know any of them. We don’t talk much to them. It’s just not something we do as students. They seem unreachable in that way, like you would be crossing a line if you tried. All students think that way (Robert, Biology, Lower educational family background, UQ).

They (educators) were like heroes we couldn’t ever speak to. It was just like they came from a different planet, or were like rock stars. Everyone would really like to approach them and discuss the topic but they were in our head unreachable. So distant to us, we just wouldn’t do that. Nobody does (Nina, Psychology, Lower educational family background, AU).

I don’t think that they (educators) distance themselves on purpose. Just the signal in them standing down there lecturing and we are sitting here passive – just listening… that way they come to seem very special, they are of course, they are lecturers and they are very skilled. But it’s just like students inherit this unspoken rule from somewhere about a border that we are students and they are something else and we don’t interact (Niels, Nano Science, Lower educational family background, AU).

The following descriptions are from students from higher educational family backgrounds.

We don’t really talk to the educators. It’s like we just know that we can’t waste their time with our silly questions. It’s just the way it is. It is like this in all courses…I guess we just know that as students. I think most students even get annoyed by other students that do not seem to be aware of this. Because we need the lecturers to talk to teach us, and not students using up the little time we have with them (Jacinta, Psychology, Higher educational family background, UQ).

We hardly ever contact educators, there is just a big gap, and when we have lectures it is just common knowledge among students that we don’t ask a lot of questions. We have such a short time to hear them talk about things that it is just not something we do. If some students do that they are sent a lot of stares and sighs from other students, until they stop doing it. And also you don’t just walk up to their (educator’s) office, because we are just students and they are real professionals (Marlene, Medicine, Higher educational family background, AU).

In conclusion, regardless of family background, it appears that strong bonding social capital with other students may hinder other forms of social interaction that have the potential to give rise to identity formation.

### THE AVAILABILITY OF BRIDGING SOCIAL INTERACTIONS

The process of professional identity formation was not only hindered due to students’ own bonding social capital preventing them from approaching educators during lectures or classes, it was further complicated by the difficulty in finding occasions to engage in social interaction that could help them gain bridging social capital. The first extracts are from students from lower educational family backgrounds.

We do need interaction with educators to understand how to use this learning, but something prevents it…I’m just not good at taking such initiatives when I feel a bit out of place. I think that we (students) all just feel that they are very different from us…that they belong to something that we still don’t (belong to). It’s not a barrier that you just ignore (Damien, Natural Science, Lower educational family background, UQ).

I really need more support in finding out who I am as a professional. I can only gain that from educators here. But we don’t have much interaction so it is difficult…maybe I should be better at approaching them, maybe I just don’t do that enough, but I feel like I go out of my place if I do that (Erica, Psychology, Lower educational family background, UQ).

I think that it is so difficult becoming a professional. I wish I felt better about that whole side of me. I try to be very alert, when listening to them speak (educators). I wish I could just go up and talk to them and felt ok about that. I should maybe do that…I just feel that is wrong doing that. I’m not good at approaching people like that. They are way out of my league. So I don’t. I don’t feel like I can do that when I’m still just a student (Gine, Political Science, Lower educational family background, AU).

One noteworthy observation is that students from lower educational backgrounds frequently made internal attributions for the failure to develop professional identity (“I am just not good at taking initiative,” “not good at approaching people”). In contrast, there was a tendency among students from higher educational backgrounds to make external attributions for such failures. The extracts below are from students from higher educational family backgrounds and they focus on the hierarchy at universities, the limited opportunities at university for interactions between students and educators and “the way this system is set up.”

We don’t have much contact with professors and lecturers and so on, because they are of course at the highest level, where they write articles and do research and so on. Generally, we only have contact with a few clinical educators. This environment does not support a lot of contact between us. There are great distances in hierarchy within the academic field here at university. And educators belong to something else than us…we are just students. So we don’t just meet (Soren, Odontology, Higher educational family background, AU).

I don’t find much social interaction with educators… like lecturers they seem really far away sometimes, it’s like interactions between students and educators is too far to bridge for either part…And there are not really any opportunities to talk or interact the way academic learning is set up in this environment We seem to belong to different groups and we don’t really go out and play together, as there are no opportunities to do that. I guess it’s that simple (Cecilie, Psychology, Higher educational family background, AU).

I wish that I knew more about me as a professional. That would support me so much in being here... I really need contact with educators to really get a grip on that, but there are not many opportunities to have such contact. The way this system is set up we hardly know any of our educators and they certainly do not know us. In my earlier experiences it was easier, because I knew my teachers and they knew me. They said things like…you can be this or this with your skills, I will support you in this… and so on, but no one gives that form of support here. That is so hard not having that, and that no one is supporting you in where you’re going (Alexandria, Business Communication, Higher educational family background, UQ).

In sum, regardless of family background, it was difficult for all students to create occasions for bridging social interaction with educators. This also meant that professional identity formation was difficult for all students. However, there was a tendency for students from lower educational family background to make internal attributions whereas students of higher educational family backgrounds made external attributions for their failure to interact with educators.

## DISCUSSION

We examined within two university contexts whether different forms of social capital facilitate academic and professional identity formation. In a nutshell, we found that social capital formation is an ongoing process that continues to affect identity formation after students have entered university. Despite the differences between the universities and the different educational contexts in these two countries, experiences across the two contexts were rather similar. This suggests that event though there are many differences between universities, these differences in structure, size, funding, educational philosophy, to name just a few, did not appear to shape students’ experiences differently. Indeed, it appears that the difficulty of identity formation is a process that was encountered by all students to the same extent and that these issues came to the fore not at a macro-level but in students’ interpersonal interactions with other students and with educators.

Interestingly too, students’ family background did continue to impact on their identity formation albeit in different ways than described in the literature. Specifically, previous findings suggest that engagement in extra-curricular activities provide, in particular for students from lower SES backgrounds, bonding capital opportunities that facilitate academic identity (Astin, 1993; Tinto, 1993; Pascarella and Terenzini, 2005; Kuh et al., 2006; Gardner and Barnes, 2007). In contrast to this previous work, even though we found that bonding social capital with co-students was perceived as beneficial for academic identity formation, students described social interactions relating to academic learning and the bonding capital they gained from such interactions as better facilitators of academic identity formation than interaction opportunities they had with co-students during extra-curricular activities.

What is more, while students from higher educational backgrounds seemed interested in such activities, several students from lower SES background mentioned that they did not enjoy participating in extra curricular activities. This suggests that offering extra curricular activities at university in an attempt to resolve identity issues may not be the answer for students from all backgrounds. In effect, such activities may foster continued inequality in opportunities on the basis of background. A more promising way to build bonding capital would be to consider ways to enhance opportunities for group based interactions, and in particular one-on-one interactions related to academic learning. Regardless of parents’ educational background, students perceived this to be the most beneficial form of bonding social capital in facilitating academic identity formation.

Interestingly too, regardless of educational family background, we did not find any evidence that students gained bridging social capital from social interaction with co-students. This finding is at odds with previous research which has suggested that in particular minority group students can gain social capital by engaging with majority group students or higher SES students because this enhances their sense of belonging within the academic environment (Thomas, 2002; Scanlon et al., 2007). We can only speculate why we did not replicate these previous findings and suggest that because all students, regardless of educational family background, felt insecure about their academic identity, sharing experiences with others from the same or a different background may not have facilitated the development of bridging capital.

Even though bonding social capital with co-students that related to academic learning was perceived as the most effective form of bonding social capital, the social bonding capital that emerged from these interactions did not facilitate academic identity formation to a significant extent. Bonding social capital with co-students mostly enhanced students’ feeling of belonging to the academic environment and supported student retention by creating a safety net that prevented students from dropping out. This finding is consistent with the work by Archer (2008) who also calls attention to the many setbacks that students experience and that make them insecure and uncertain of whether they should continue their studies. The bonding social capital found in relation to academic learning may be a source of helpful support to cope with these fears.

There were also downsides to this bonding social capital. Bonding social capital also created homogeneous student groups that developed their own distinct group norms, with some of these norms prescribing members to not seek out contact with educators, thereby hindering the formation of bridging capital with educators. This finding is consistent with Putnam’s observation that bonding social capital can be restricting and limiting because the strong social control within these social networks can limit its members’ freedom of action (Putnam, 2000). There is also evidence that a lack of social interactions between students and educators negatively affects retention (Tinto, 1993; Dowd and Korn, 2005; Kuh et al., 2006; Scanlon et al., 2007) and our findings show that the lack of such contact also negatively affects the formation of academic and professional identity. Our results indicate that students may cope with the challenges of university life by bonding with co-students. Even though this is associated with beneficial effects for students, this may not help them to develop their identities to their fullest, especially their professional identities, or achieve their highest academic learning outcome possible.

In this study we found that students’ bridging social capital with educators was the facilitator in students’ academic and professional identity formation. Unfortunately all students felt that bridging social capital was difficult to obtain and therefore professional identity was difficult to develop. Interestingly, students’ parental background did not affect the perceived difficulty of forming professional identity. In the countries represented in this study, educational opportunities are fairly open to all students. However, there was a tendency for students from lower educational backgrounds to be more likely to make internal attributions for the failure to develop professional identity while those from higher SES backgrounds were more likely to make external attributions for such failures.

Difficulty in forming academic identity and professional identity in particular, may therefore be experienced for different reasons. It could be that high SES students are generally used to more contact with educators than low SES students (Calarco, 2011). High SES students may therefore be more vulnerable when they experience a gap in student—educator contact and when they do not get the same level of attention from teachers as they were used to receiving in high school. This can leave high SES students vulnerable because they are not prepared for such a situation. In contrast, even though low SES students may be used to less contact and support and therefore may be more resilient in this situation (Kim and Sax, 2009; Calarco, 2011), they too may not have the means to obtain bridging social capital with educators and they may attribute this to lack of personal communication skills or they may associate it with cultural norms (Stephens et al., 2014).

Regardless of the specific reason for the difference in attributional style by educational family background, it is clear that external attributions for the failure to develop professional identity are more self-protective than internal attributions for such failure. Furthermore, attributional style differences can contribute to the maintenance of social class inequalities in that it is higher SES students who will demand better a better service by the educational system whereas lower SES students will not push for structural change to improve opportunities to develop professional identity.

Our results suggest that to overcome such barriers, students from both lower and higher educational family backgrounds may benefit from social interaction opportunities that are planned around academic activities. These should not just focus on fostering bonding interaction among students but also on creating opportunities for the development of bridging social capital, which, in turn, is essential for students’ professional identity development.

### Conflict of Interest Statement

The authors declare that the research was conducted in the absence of any commercial or financial relationships that could be construed as a potential conflict of interest.
